# Fluorescence Invigoration in Carbon-Incorporated Zinc Oxide Nanowires from Passage of Field Emission Electrons

**DOI:** 10.1038/s41598-019-46177-w

**Published:** 2019-07-04

**Authors:** Andrew Bah, Kim Yong Lim, Fuhua Wei, Anjam Khursheed, Chorng Haur Sow

**Affiliations:** 1 Department of Physics, National University of Singapore, 2 Science Drive 3, Singapore, 117542 Singapore; 2 Department of Electrical and Computer Engineering, National University of Singapore, 4 Engineering Drive 3, Singapore, 117583 Singapore

**Keywords:** Nanowires, Nanowires

## Abstract

We demonstrate that carbon incorporated Zinc Oxide (C-ZnO) nanowires (NWs) exhibit remarkable improvement in the extent and quality of fluorescence emission after they are utilized as an electron source in a field emission experiment. After the passage of field emission electrons, the intensity of the fluorescence emitted from these NWs in the visible light range exhibits a 2.5 to 8 fold enhancement. The intrinsic exciton peak of the ZnO also becomes heightened, along with the crystalline quality of the NWs showing marked improvement. This invigoration of fluorescence across the entire fluorescence spectrum is attributed to concurrent removal of oxygen and carbon atoms in C-ZnO NWs due to electro-migration of atoms and joule heating during the field emission process. Applications based on ZnO NWs emission from excitonic emissions or visible wavelength emissions or both can benefit from this straightforward method of defect engineering.

## Introduction

Zinc oxide (ZnO) typically has a direct band gap of 3.37 eV. In a photoluminescence (PL) scan, ZnO exhibits a characteristic peak at around 380 nm corresponding to the exciton recombination. In addition, a broader peak within the visible-IR region is often observed and has been attributed to emissions from defect sites in the crystalline structure of the ZnO and is commonly known as the defect band^1^. This defect band usually comprises of several different peaks, each of which arises from a different species of ZnO crystal defects^1–3^. The ‘peak wavelength’ of the defect band will shift depending on which defect type is more dominant in the ZnO crystal structure. While the fluorescence of ZnO has been widely studied and many of the defect-associated emissions have been documented, there are still some under investigation^4^. Some of the reported values for the various peaks and their respective origins are ~530 nm ‘green’ peak due to oxygen vacancies or zinc vacancies^3,5–7^, ~630 nm ‘orange’ peak due to interstitial oxygen, 730 nm ‘red-orange’ peak due to carbon incorporation in ZnO etc^8^.

The field emission (FE) capabilities of ZnO nanostructures has also been a subject of extensive research interest. The variety of nanostructures that can be grown from ZnO has spawned much research into field emission capabilities of ZnO nanorods, nanopins, belts, films, as well as the effects of ordered arrays, doping and substrate choice^9^. However, to the best of our knowledge, there has been little work on how the fluorescence of the ZnO nanowires (NWs) is affected by the use of these NWs as field emitters. Most of the previous work also implicitly assumes that the ZnO NWs field emitters are not altered in the field emission process. In addition, while ZnO nanostructures’ field emission properties have been extensively studied, similar data on carbon incorporated ZnO (C-ZnO) NWs are presently unavailable. As highlighted in previous reports, the heavy doping of carbon into the ZnO system can give rise to optical^10,11^ and magnetic states unavailable to pure ZnO nanostructures^12^. This offers possible applications of C-ZnO nanostructures in the areas of light emitting diodes^10^, photo-catalytic^13,14^ and gas sensing applications^15^. This myriad of applications stand to benefit from an understanding of how field emission as a post-growth processing tool can provide a viable path for modification and engineering of ZnO and carbon incorporated ZnO nanowires.

In this work, we demonstrate that ZnO NWs can exhibit remarkable improvement in the quality of fluorescence emission after they are utilised as an electron source in a field emission experiment. The intensity of fluorescence emitted from these NWs in the visible light range and the NWs’ crystalline quality are shown to be enhanced after field emission. The investigation of this invigoration of fluorescence points to field emission currents removing oxygen and carbon-related defects in ZnO and C-ZnO NWs, thereby changing the optical properties of the NWs.

The carbon-enriched ZnO NWs used in this work are grown by a chemical vapour deposition (CVD) process following the method (Fig. A1) described previously^8,16^. Samples undergoing field emission are placed in a typical parallel-plate configuration field emission setup as shown in Supplementary Information (Fig. A2). Zinc Oxide NWs with defects that emit in green fluorescence under UV illumination and C-incorporated ZnO NWs with defects that emit orange-red fluorescence are subjected to field emission in this work. To quantify the performance of the samples as field emitters and allow comparison with other such work, we make use of two standards. The “turn-on field” is the electric field required to produce a field emission current density of 0.1 *µ*A/cm^2^, and the “threshold field” is the electric field required to produce a current density of 1 mA/cm^2^ from our samples.

The effects of field emission process on both types of ZnO based NWs are examined with fluorescence microscopy (FM), photoluminescence (PL) and X-ray Photoelectron Spectroscopy (XPS). The striking changes in fluorescence behaviour in both samples after field emission are observed and quantified. Comparison with Kelvin Probe force Microscopy (KPFM) data also helps to explain the differences in field emission data of both forms of ZnO–based NWs. This work also illustrates the potential of modifying and even enhancing the fluorescence of ZnO-based NWs by means of electrical currents.

## Results and Discussion

### Fluorescence modification via field emission

Following the growth technique as described previously^8^, ZnO NWs that emit different fluorescence colours under UV excitation are first grown by varying the growth parameters^8^. The NWs grown at 900 °C emit green fluorescence under UV excitation whereas the NWs grown with the zinc oxide/carbon source temperature at 850 °C emit orange-red fluorescence under UV excitation.

**Figure 1:**
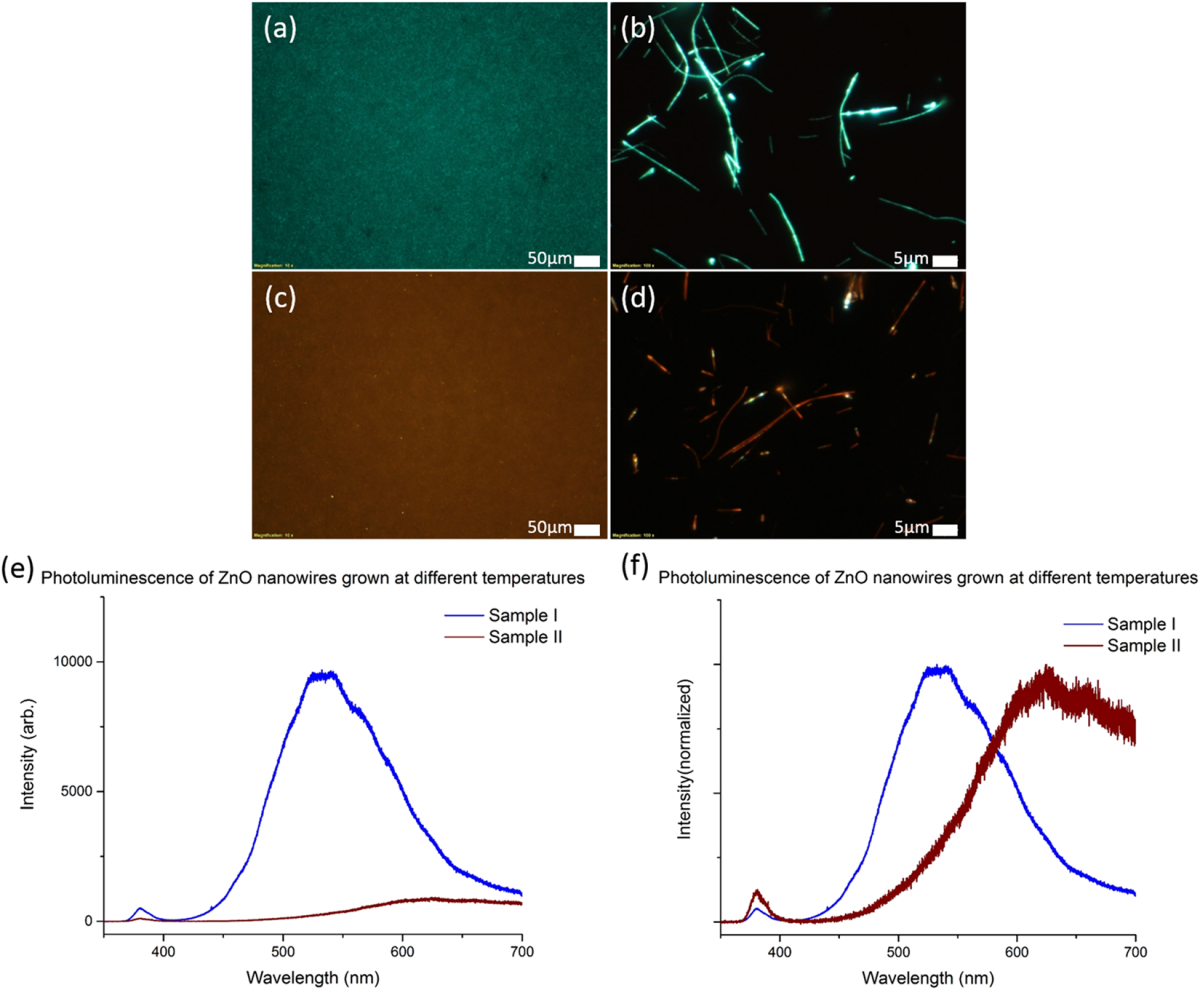
UV Fluorescence microscope images of Sample I (**a**,**b**) and Sample II (**c**,**d**). (**a**,**c**) Show the as-grown samples under low magnification (scale bar = 50 *µ*m), while (**b**,**d**) are of individual NWs after a dry contact transfer process to silicon wafers (scale bar = 5 *µ*m). Graph (**e**) shows the photoluminescence spectra for Sample I and II, highlighting the difference in fluorescence intensity of the two samples. Graph (**f**) plots the normalized PL data for the two samples, with Sample I peaking at 535 nm, and Sample II peaking at 650 nm.

From here on, the sample exhibiting green fluorescence is labelled as Sample I and the sample exhibiting orange-red fluorescence is labelled as Sample II. Figure 1 shows fluorescence microscopy (FM) images of the ZnO NWs studied in this work. High density NWs are grown on a silicon (Si) substrate. Figure 1(a–d) show top view FM images of dense as-grown NWs array samples I and II respectively. The distinct green fluorescence of sample I (Fig. 1(a)) and orange-red fluorescence of sample II (Fig. 1(c)) under UV illumination are clearly observed. Individual NWs are then transferred to the silicon substrate via a dry contact transfer method. The FM images of the NWs from sample I and II under UV illumination are shown in Fig. 1(b,d) respectively.

Close examination of individual NWs reveal that the NWs from Sample I appear homogeneously green in fluorescence with some variation in the intensity produced by individual NWs. The NWs from Sample II on the other hand are dominantly orange-red in fluorescence with occasional spots of yellow-green fluorescence. The overall macroscopic fluorescence intensity of Sample I is also noted to be higher than that of Sample II.

The photoluminescence (PL) data comparison for the ZnO NWs grown at the two different temperatures shows the difference in peak positions consistent with observations from FM images. In the PL spectra for the two samples (Fig. 1(e)), Sample I presents a fluorescence band around 535 nm (i.e. green fluorescence) and the PL intensity of the orange-red sample is significantly lower compared to the green fluorescence. In Fig. 1(f), the normalized PL spectra shows that Sample II presents a fluorescence band that is centred at 650 nm, giving rise to the observed orange-red fluorescence. As reported, Sample I ZnO NWs produce a typical green fluorescence under UV excitation with the defect peak centred around 525–550 nm^17^. The green fluorescence is often attributed to oxygen vacancies in ZnO. Sample II NWs are considered to be C-ZnO NWs resulting from the off equilibrium growth with carbon incorporation in the ZnO NWs^8^.

**Figure 2:**
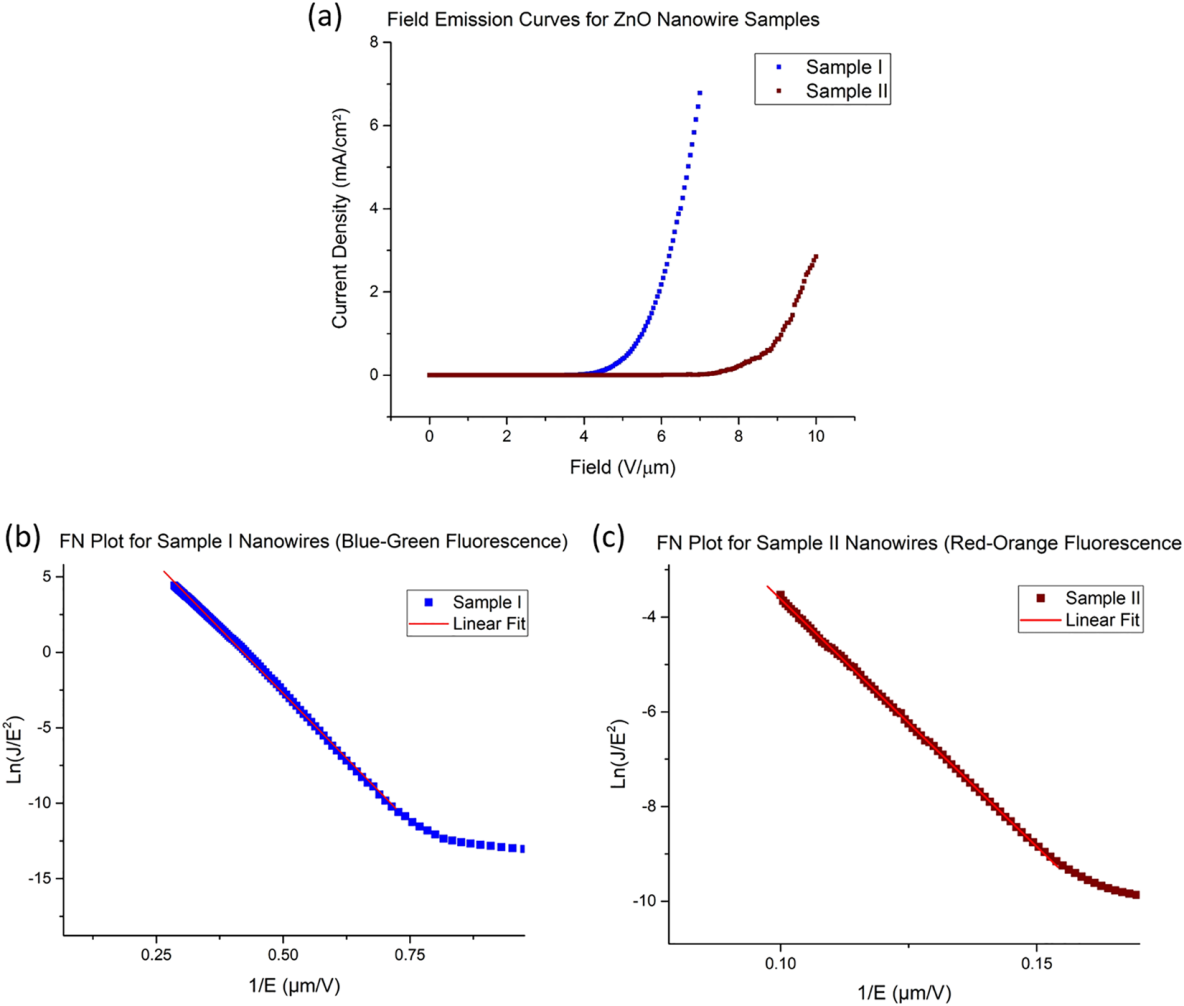
(**a**) Field emission I-V plots for Sample I and II. Turn-on and threshold fields for Sample I: 3.85 *V*/*µm* & 5.55 *V*/*µm*, Sample II: 6.90 *V*/*µm* & 9.15 *V*/*µm*. Fowler-Nordheim (F-N) plots are shown in (**b**) for Sample I and in (**c**) for Sample II NWs.

Figure 2 shows the characteristic curves of the samples in field emission measurements after 10 field emission cycles. Sample I ZnO NWs has a turn-on field of 3.85 *V*/*µm* and a threshold field of 5.55 *V*/*µm*. On the other hand, the turn-on field for Sample II is found to be 6.90 *V*/*µm* and its threshold field is measured to be 9.15 *V*/*µm* (Fig. 2(a)). Some published values for the turn-on and threshold field for ZnO NWs are 2.5 *V*/*µm* and 4.0 *V*/*µm*^18^, 6.0 *V*/*µm* and 11.0 *V*/*µm*^19^, 7.0 *V*/*µm* and 17.8 *V*/*µm* respectively^20^. Our samples’ performance as field emitters are comparable to published values, with Sample I NWs being better field emitters than Sample II NWs. Fowler-Nordheim (F-N) plots of Sample I and Sample II are shown in Fig. 2(b,c) respectively. The F-N plots exhibit linear characteristics in the high electric field regime, characteristic of the field emitting nature of the nanowires.

**Figure 3:**
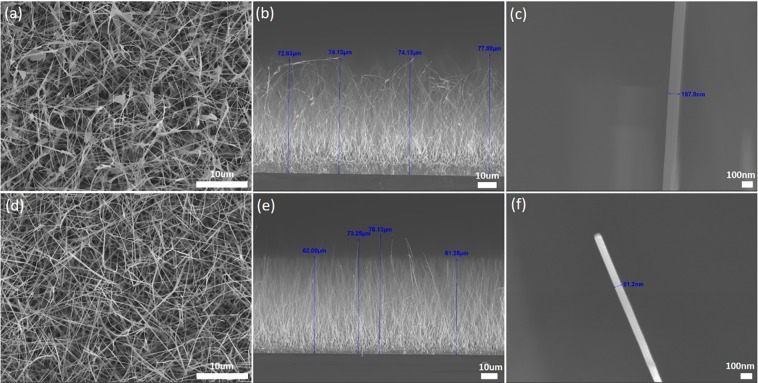
SEM images of Sample I and II in (**a**,**d**) as viewed top down; (**b**,**e**) as viewed edge-on; and (**c**,**f**) as viewed individual NWs respectively. As seen from (**b**), Sample I NWs lack the uniform directionality and length in contrast to Sample II NWs in (**e**). However the maximum height of the NWs as measured from the edge view of both samples are comparable at 75–80 *µ*m. Diameter of the individual NWs at the top of the samples was found to range from 80–120 nm in both samples. Estimates of the tip density for NWs near the top of the samples where field emission is expected to occur were calculated to be 0.75 ± 0.05/*µm*^2^ and 0.82 ± 0.05/*µm*^2^ for Sample I and II respectively.

In relation to the field emission capability of nanowire forests, geometry and field emitter density are important considerations. Figure 3(a,d) show the top-down view of Samples I and II respectively and both appear to be similar. However, the side profile SEM images, in Fig. 3(b,e) for Sample 1 and Sample 2 respectively, display differences in the nanowire growth of the two samples. While growth in both samples are largely along the vertical direction, Sample II NWs are more aligned and uniform in height and appear to be more densely packed compared to Sample I NWs. Both samples have a maximum nanowire height of 75–80 *µ*m, with the individual diameters of the NWs falling in the range of 60–110 nm. Figure 3(c,f) display the respective close-up images of a typical NW in Sample I and Sample II. Due to the nature of Sample I NWs, their individual lengths exceed that of the height of the NWs arrays. As the NWs of Sample II are more densely packed near the tips, we expect the shielding effect of the electric field to be greater compared to Sample I. These variations can confound and complicate efforts to evaluate the NWs’ work functions and the field enhancement factors from the field emission characteristic curves alone.

**Figure 4:**
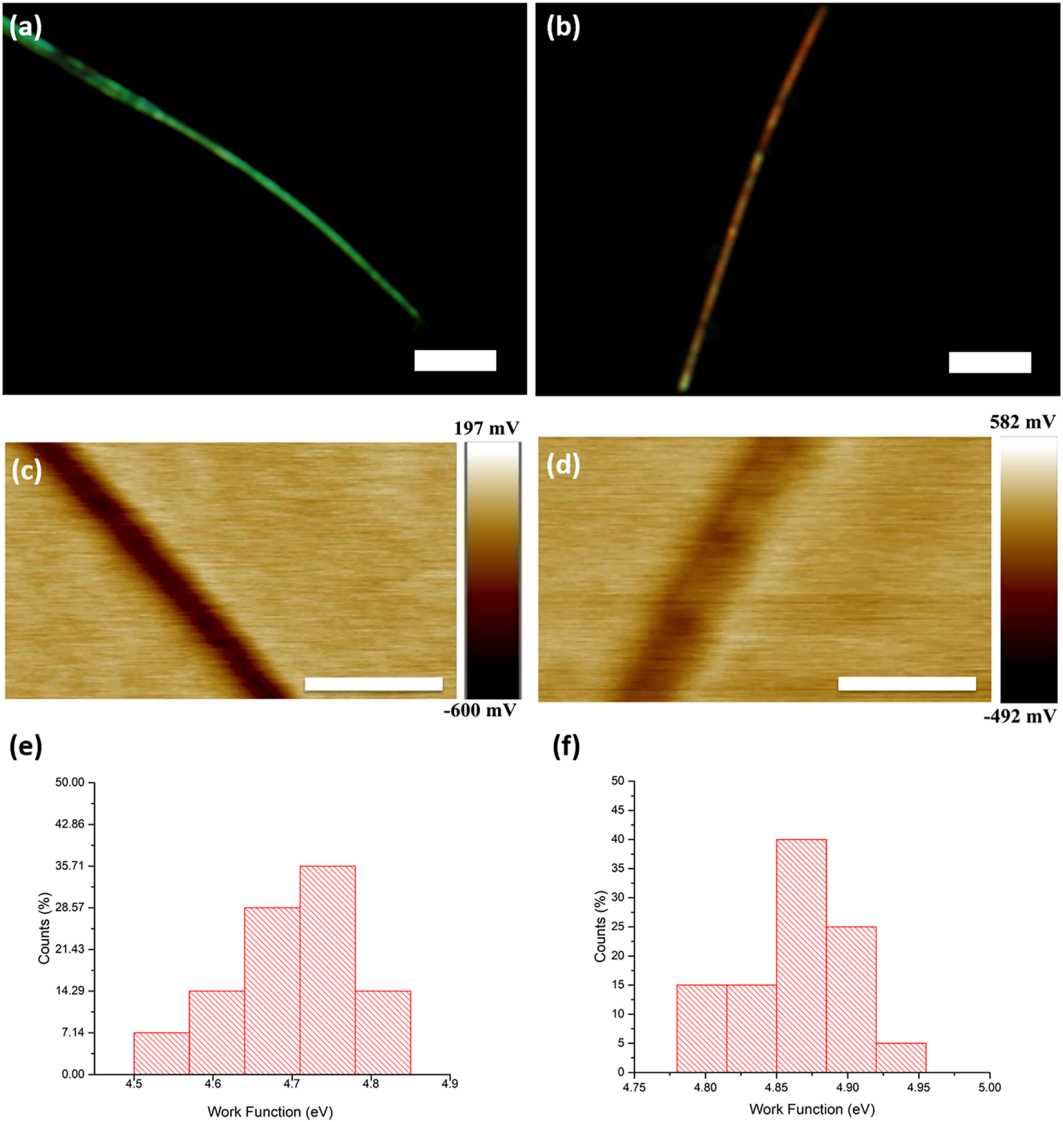
FM images of individual NWs of Sample I (**a**) and Sample II (**b**) and their corresponding FM-KPFM scans (**c**,**d**). Measured with respect to the work function of a gold substrate, the calculated work function values for the NWs are 4.65 ± 0.07 eV for green fluorescing NWs (Sample I), and 4.85 ± 0.07 eV for red-orange fluorescing NWs (Sample II). (**e**) Distribution of work functions of green fluorescing ZnO. (**f**) Distribution of work functions of red-orange fluorescing ZnO. (scale bar = 10μm in (**a**) and (**b**); scale bar = 500 nm in (**c**) and (**d**)).

In order to over-come the above-stated problems and to assess the field enhancement factor due to the nanowire geometry of the two samples, the work function of the NWs in both samples are first measured using Kelvin Probe Force Microscopy (KPFM). The KPFM is used to measure local surface potentials of NWs from which the work functions can be independently deduced^21^. Figure 4(a,c) show the FM image and the corresponding KPFM scan of a probed NW from Sample I. Figure 4(b,d) show the FM and corresponding KPFM scan of a probed nanowire from Sample II. Tens of NWs in both samples are probed and their surface potentials are measured. The respective work function distribution histograms of Samples I and II are shown in Fig. 4(e,f) respectively. Operating in the frequency modulated mode (FM-KPFM), the measured surface potential values imply work functions of 4.65 ± 0.07 eV for Sample I NWs, and 4.85 ± 0.07 eV for Sample II NWs respectively. The difference of ~0.2 eV between the two work-functions is significant and can be meaningfully compared against each other, as validated by other similar work with ZnO doped with other elements^22^.

Elucidation of the NWs’ work functions in both samples can help pinpoint the nature of defects within the NWs. Possible carbon-related defects with inter-band defect energy levels that can give rise to the respective emissions (green and orange-red) in C- ZnO have been formerly elucidated via density functional calculations^8,23^. Their highest occupied defect energy levels are illustrated in Fig. A3 (Supplementary Information). The above work function measurements impose a further constraint where the work function of orange-red fluorescing NWs (Sample II) is higher than that of green fluorescing NWs (Sample I) by ~0.2 eV. Extending this constraint to the calculated carbon-related defects, we may deduce the nature of the dominant defect within NWs with different fluorescence. As shown in Fig. A3 in the Supplementary Information, the highest occupied energy level due to each type of defects are shown, along with their corresponding work functions. Only a pair of defects with matching emissions and computed work functions satisfy the criterion imposed by the measured work-function values i.e. the 2C_o_-Zn_i_ defect is deduced to be the dominant defect for orange-red emission while the C_o_-Zn_i_ defect is the dominant defect for green emission. Additional substitution of oxygen by carbon in 2C_o_-Zn_i_ defect complex in Sample II as compared with C_o_-Zn_i_ defect complex in Sample I suggests that Sample II have a more abundant concentration of carbon than in Sample I.

Following from these work-function measurements, we further employ a simplified Fowler- Nordheim type equation to the field emission data for the computation of enhancement factors of the two samples

$$J(F)=(\frac{A{F}^{2}}{{\rm{\Phi }}})\exp (\frac{-B{{\rm{\Phi }}}^{\frac{3}{2}}}{F})$$

with enhanced field *F* = *βE* with *β* being the enhancement factor. From the data fit of our F-N plots in Fig. 2(b,c) and the measured work functions, we calculate the values of the enhancement factors for the two samples to be 1990 ± 50 and 700 ± 10 for Samples I and II respectively. From these values, we argue that even though Sample II NWs are well aligned with tips facing the cathode, the shielding effect due to the dense packing of the NWs results in a significantly lower electric field enhancement factor.

**Figure 5:**
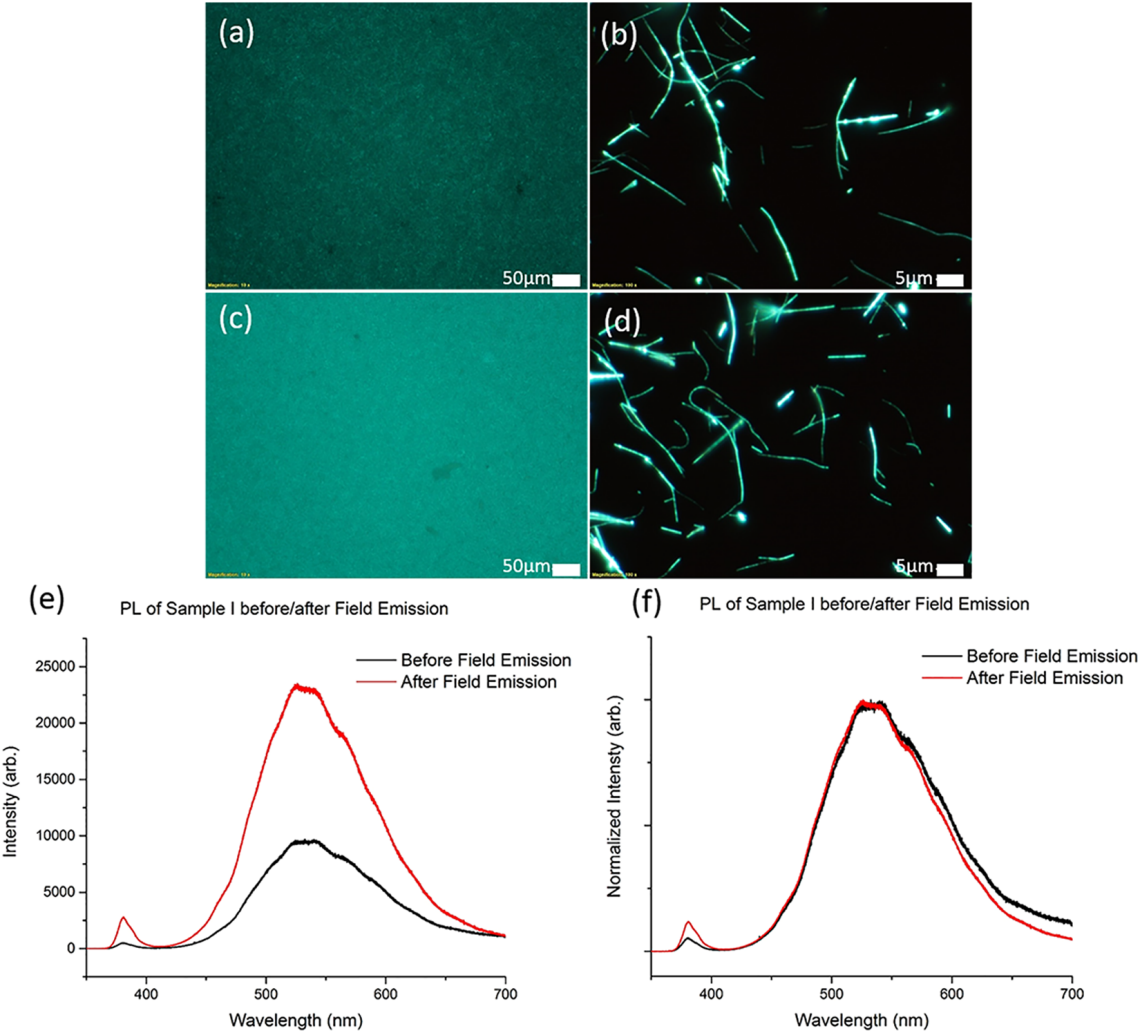
FM images of Sample I (**a**,**b**) before field emission and (**c**,**d**) after field emission. (**c**) Shows that the sample exhibits a higher fluorescence intensity after field emission as compared to (**a**). Graph (**e**) is the PL plot of Sample I which shows a 3 times increase in defect band intensity after field emission. Graph (**f**) is the normalized PL plot of Sample I showing a slight decrease in the width of the defect peak as well as an increase in exciton peak-to-defect peak ratio after field emission.

**Figure 6:**
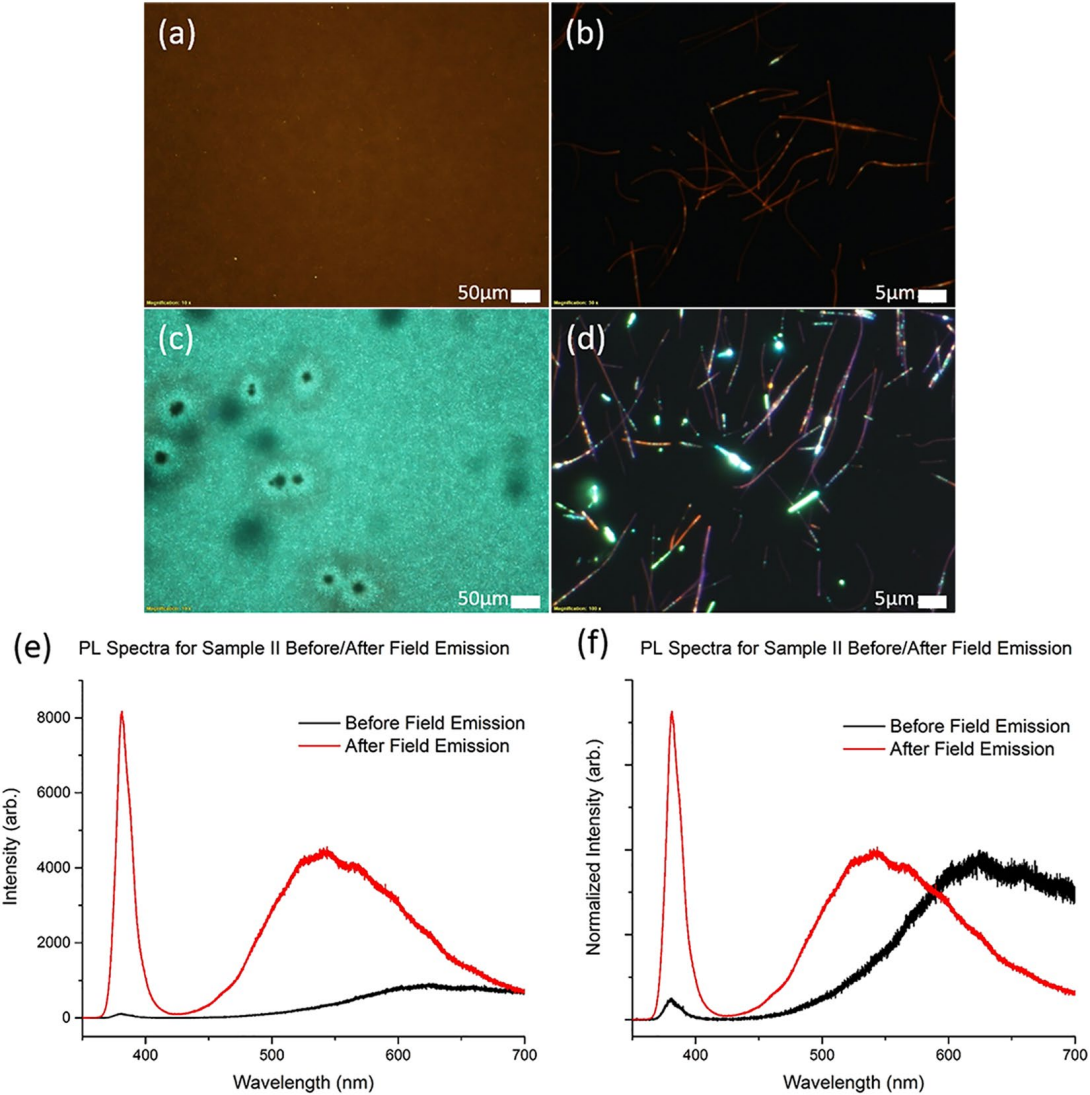
FM images of Sample II (**a**,**b**) before field emission and (**c**,**d**) after field emission. Graph (**e**) is the PL plot of Sample II showing an 8 times increase in intensity of the defect peak after field emission. Graph (**f**) is the normalized PL plot of Sample II showing a blue shift in the defect peak from around 650 nm to 535 nm as well as a significant increase in the exciton-to-defect peak ratio after field emission.

After the samples are tested as field emitters for 2 hours at their threshold fields, their fluorescence properties are re-evaluated and discovered to be altered and significantly improved. Two points of interest are observed. Firstly, it is found that the emission intensity of the samples are increased by a significant amount after being used as field emitters. Figure 5(a,c) shows the FM images of Sample I before and after field emission respectively. Figure 6(a,c) shows the FM images of Sample II before and after field emission respectively. From the PL data, the integrated area of the defect peak after field emission shows an increase by almost 3 times for Sample I, and almost 8 times for Sample II, as noted in Figs 5(e) and 6(e) respectively.

FM images of the individual NWs, Fig. 5(b,d), do not immediately show significant changes in the individual nanowire fluorescence but the cumulative effect is only apparent when observing the sample at low magnification. The increase in fluorescence intensity remains after the sample is left under ambient conditions over several days. This suggests that the change in fluorescence is due to a chemical change, rather than just the temporary removal of surface adsorbed contaminants.

Since visible range fluorescence is attributed to the defects in the ZnO lattice, this increase in fluorescence would indicate an increase in the density of defect sites. However, we also note that the exciton peak to defect peak ratio shows an increase after the field emission process. There is also a slight narrowing in the spread of the defect peak around the 600–700 nm region of the spectra as seen in Fig. 5(f), suggesting a reduction in C-incorporated species believed to be responsible for the red-orange emission colour.

Secondly, the fluorescence colour of Sample II is found to demonstrate a significant transformation, changing from orange-red to green. This change results in Sample II, initially having an orange-red fluorescence, to having a post-field emission fluorescence similar to that of Sample I. As shown in Fig. 6(a,c), the region that has undergone field emission in Sample II changes from fluorescing orange-red to green. The NWs forest appear to be uniformly green when seen under low magnification. However, upon observation at high magnification, the individual NWs show that the green fluorescence are emitted from small regions of high intensity along the NWs as shown in Fig. 6(d). This is in contrast to Fig. 6(b) where the C-ZnO NWs appear largely orange-red and hence laden with carbon-related defects. The rest of the nanowire is fluorescing orange-red or violet under UV excitation. This is consistent with the photoluminescence data for Sample II in Fig. 6(e,f) which shows the blue shift of the defect peak indicative of that observed in the FM images. The fluorescence colour of the sample and the position of the defect peak at around 535 nm after field emission also matches that of Sample I. The increase in intensity of the green fluorescence seen in Fig. 6(d) suggests that the field emission process causes the density of the oxygen defects in some of the NWs to increase.

Simultaneously, the ratio of the 380 nm exciton peak to the defect peak was also found to have significantly increased after the field emission process for Sample II, partially manifested as the presence of violet coloured NWs in the FM images. Hence, the NWs appear to have undergone improvements in lattice quality, as evidenced by the increase of the exciton peak to defect peak ratio. The change in the peak ratios of the PL of Sample II before and after field emission could be caused by the passage of field emission electrons that drive away the carbon-related defect species. This leaves behind the higher quality ZnO nanowires, which thus increases the exciton peak to defect peak ratio.

As the NW is originally laden with carbon-related defects in various forms and also possibly in less ordered form, the removal of these defects by joule heating etc. means that there are less scattering centres and energy losses by phononic processes are reduced. This allows for crystals with more order, even if defects such as oxygen vacancies exist. It is noteworthy that we have not observed a sharpening of the NWs nor any significant increase in the sharp edges in our samples after field emission. This eliminates the possibility of an increase in vertical field emission points.

### X-ray photoelectron spectroscopy

**Figure 7:**
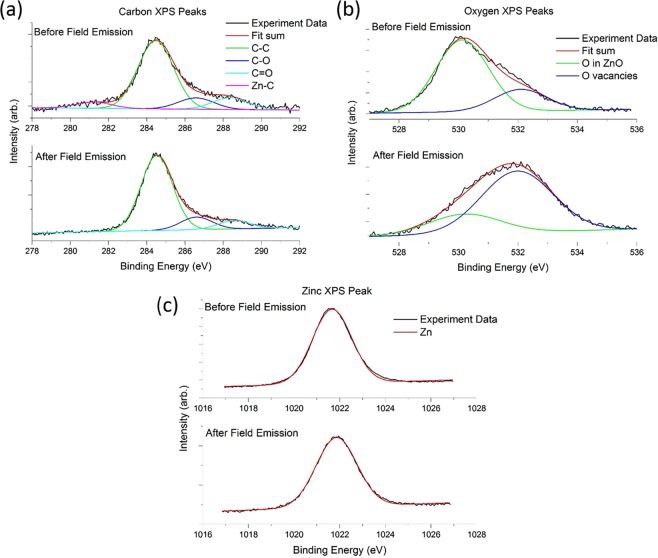
XPS scans for Sample II (red-orange fluorescence) before and after field emission. (**a**) Carbon XPS peaks show the suppression of a peak associated with Zn-C bonds after field emission. (**b**) Oxygen XPS peaks show the increase in peak associated with oxygen vacancies in the ZnO lattice after field emission. (**c**) Zinc XPS peak shows a slight shift to higher binding energy after field emission.

To verify the above inferences, X-ray photoelectron spectroscopy (XPS) is performed on Sample II to assess any changes to the chemical bonding or composition of the NWs after being used as a field electron emitter. The XPS spectra are presented in Fig. 7. The XPS scan of the 280–290 eV binding energy region for Sample II (Fig. 7(a)) before undergoing field emission indicates the presence of adventitious carbon C–C bonding (284.6 eV), C–OH bonding (286.5 eV), C=O bonding (288.1 eV)^24^ as well as a peak around 281.2 eV which is thought to be attributed to Zn-C bonding^8^. After the use of Sample II as a field emitter, we find that the peak associated with Zn-C bonding has been suppressed.

The XPS spectrum of Sample II for the oxygen region, Fig. 7(b), gives rise to a peak at 530 eV attributed to the O^2−^ ions of the wurtzite structure in ZnO when surrounded by a full complement of Zn^2+^ and O^2−^ ions^8,25^. A second peak at 532 eV is associated with O^2−^ ions in oxygen deficient regions of the ZnO matrix^8,25^. The ratio of the areas subtended by the peak attributed to the oxygen in ZnO to the peak associated with the oxygen vacancies in the lattice changes from 1:0.30 (before field emission) to 1:3.1 (after field emission). This suggests a large increase in oxygen vacancies and is in line with the PL results for Sample II, which show an increase in fluorescence in the 525 nm region, often reported to be due to oxygen vacancies in ZnO^7,26,27^. The Zn XPS spectrum of Sample II in Fig. 7(c) shows a slight shift to higher binding energy for the peak at 1022 eV which we believe to be within the margin of error for our measurements. The suppression of the peak associated with Zn-C bonds, as well as the increase in the peak for oxygen vacancies are indicative of a process involving the removal of both carbon and oxygen from the C-ZnO NWs during the field emission process. It is to be noted that XPS has a probe depth within a few nanometers of the surface.

C-ZnO NWs have been previously shown to be less stable than ZnO NWs. The former is easier modified by thermal annealing, focused electron beam^8^ and by focused laser beam heating^28^. The supply of energy from electrical currents flowing through the NWs during field emission are likely akin to these processes which would involve electro-migration of atoms, joule heating or both.

Joule heating has been observed in field emission by carbon nanotubes^29,30^, resulting in temperature of ~2000K and is an important consideration in field emission stability of ZnO nanomaterials^31^. ZnO has a low heat conduction coefficient and this gives rise to a rapid local annealing process. Concurrently, electro-migration of carbon atoms is also likely because of the high number of such defect states within the nanowires^32^. These crystallographic defects in C-ZnO cause lattice distortions, resulting in atoms in less stable equilibrium positions and with lower activation energies, as aforementioned^8,28^. Hence, atoms are more likely to be displaced and allowed to diffuse when subjected to a high conducting electron flow density and high temperature. Other experiments have also shown that ZnO and C can undergo phase separation into self-assembled layers^33^. These factors point to the diffusion of carbon from the core of C-ZnO NWs followed by subsequent removal of some carbon and oxygen species from the C-ZnO NWs. The process allows for the corresponding fluorescence change as illustrated in Fig. A4. The vacuum environment of the field emission chamber would also have thermodynamically favoured such a process. As the field emission is conducted at pressures of below 1 × 10^−6^ mbar and in the absence of ambient oxygen, oxidation of carbon would require stripping oxygen from the ZnO nanowire lattice, resulting in an increase in oxygen vacancies.

To further confirm this, we perform plasma etching of the NWs’ surface on Sample II after field emission to reveal the optical properties of the NWs’ cores. The corresponding PL results are shown in Fig. A5(a). The PL intensity of the defect band from the plasma-etched NWs has largely been quenched with the excitonic peak to defect band ratio greatly enhanced as shown on Fig. A5(b). Hence, it can be deduced the green fluorescence (peak λ = 535 nm) originates from the shell of the NWs while the excitonic emission (peak λ = 380 nm) originates from the NWs cores. Correspondingly, this shows that the oxygen vacancies within the ZnO NWs’ lattice, after field emission, are limited to the NWs’ surface as shown in Fig. A4.

To this end, the mechanism is also consistent with XPS data which suggests that the surface has been transformed from C-ZnO to ZnO with more oxygen vacancies. The above proposed deductions are also consistent with the fact that both samples I and II contain carbon-related defect species as described earlier^8^, with Sample II containing a higher concentration of carbon. As such, the mechanism will allow for a more obvious change in fluorescence in Sample II i.e. from orange-red to green while the Sample I only offers an enhanced green fluorescence from an increased concentration of other defects e.g. oxygen vacancies.

### High-resolution transmission electron microscope

**Figure 8:**
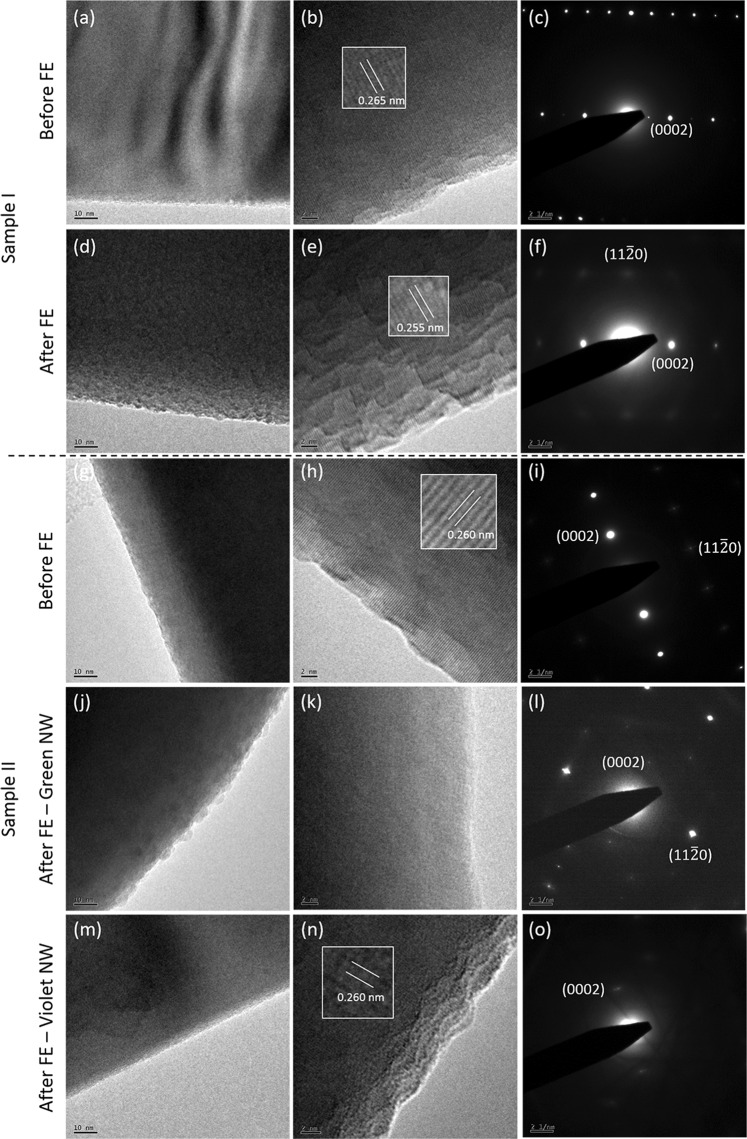
HRTEM and SAED images of ZnO NWs from Sample I (**a**–**f**) and Sample II (**g**–**l**), before and after field emission, as indicated in the left margin of pictures. Inset figures in (**b**), (**e**) and (**h**) indicate respective lattice spacing.

To trace the local source of these optical changes, Samples I and II were examined under a high resolution transmission electron microscope (HRTEM). Figure 8(a,b) are micrographs of Sample I nanowire before any field emission experiments. Figure 8(d,e) are of Sample I nanowire after field emission experiments, with Fig. 8(c,f) showing the selected area electron diffraction (SAED) pattern for the respective cases. The lattice spacing in the (0002) plane for Sample I before and after field emission decreases slightly from 0.265 nm to 0.255 nm. Also noticeable is the surface roughening of the nanowire after the field emission process (Fig. 8(e)).

Figure 8(g) to (l) are the HRTEM and SAED images of Sample II before and after the field emission process. Figure 8(g,h) shows a nanowire of Sample II before field emission. Figure 8(j,k) show NWs of Sample II after the field emission process. Figure 8(i) is the SAED pattern for a Sample II NW before field emission. Figure 8(l) is the SAED pattern for a Sample II NW after field emission, as shown in Fig. 8(j). The simultaneous presence of diffraction spots and poorly resolved diffraction rings indicates that the NW possesses both crystalline portions as well as disordered portions. Parts of the nanowire appear to have undergone a change from crystalline to a less ordered structure due to the field emission process. Nevertheless, the PL data for both Samples I and Sample II (Figs 5 and 6) show increased intensities in the defect peak and the exciton peak. It can thus be proposed that the nanowire still maintains a crystalline core that allows for excitonic radiative recombinant processes and a disordered shell layer giving rise to visible light fluorescence often associated with defects, as demonstrated earlier. The disordered outer layer could also have suppressed surface trapping processes thereby giving enhanced excitonic signatures, similar to previous reports involving argon plasma milling on ZnO NWs^34^.

### Furnace annealing

To simulate the low pressure, low oxygen and high temperature environment that the NWs experience during the field emission tests Sample II was also thermally annealed at 700 °C for 1 hour while under a constant argon flow in a low vacuum (1 × 10^−2^ mbar) tube furnace. After the furnace annealing process, the PL for the annealed sample shows a blue shift of the fluorescence from around 650 nm to 550 nm (Fig. A6). This is similar to the blue shift of the sample fluorescence observed after the field emission process seen in Fig. 6. The mechanism for the fluorescence change due to removal of carbon-related complexes from C-ZnO NWs via joule heating would therefore be similar during field emission. However, the exciton peak to defect peak ratio is slightly reduced after annealing, indicating a degradation of the ZnO lattice. This is in contrast to the after-field-emission PL data presented earlier, which indicates the field emission results in better nanowire lattice quality as shown by the increase of exciton-to-defect peak ratio after field emission. There is also no increase in fluorescence intensity for this annealed sample whereas Sample II showed a ~8-fold increase in visible wavelength fluorescence after field emission, as demonstrated in Fig. 6(e). The difference is believed to arise from the time scale in which the two processes occur. The field emission process rapidly ramps the voltage and current flow and can result in better crystallization when compared to thermal annealing^35^. This would support the PL results obtained for both Sample I (Fig. 5) and II (Fig. 6), which show an increase in the exciton peak after the field emission process. The above experiments show that field emission is capable of re-invigorating fluorescence behaviour across the NWs’ entire spectrum of optical emission or fluorescence. This phenomenon is distinct from previously discussed methods such as ambient annealing^8^, plasma treatment^8,34^ and annealing in argon which promotes fluorescence enhancement only at certain wavelength range.

## Conclusion

This work demonstrates that carbon incorporated ZnO NWs can undergo remarkable re-invigoration in PL intensity and fluorescence when used as field electron emitters. Using this method, we are able to improve the crystalline quality of the NWs as well as increase the NWs’ excitonic emissions and fluorescence. Orange-red fluorescing ZnO NWs grown through a temperature sensitive CVD process are particularly susceptible to changes in fluorescence colour due to the field emission process, as compared to green fluorescing ZnO NWs. The change in fluorescence could have resulted from electro-migration, joule heating or a combination of both during the field emission process. The process removes carbon from the NWs, causing both conversion to and enhancement of green fluorescence that is commonly attributed to oxygen vacancies in ZnO. Applications based on ZnO NWs emission from excitonic emissions and visible wavelength emissions or those that require both simultaneously can likely benefit from this straightforward method of defect engineering.

## Methods

### Synthesis of ZnO:C NWs

ZnO NWs are synthesized on a substrate with a 200 nm thick ZnO seed layer sputtered onto Si wafer by RF magnetron sputtering. The sputtering was done with 100 W RF power under an argon flow of 7 sccm, at a pressure of 1 × 10^−2^ torr and a temperature of 500 °C. A Denton Discovery-18 sputtering system was used.

To grow the ZnO NWs samples, a precursor consisting of 0.4 g of a mixture of zinc oxide (purity 99.99%) and graphite powder (1:1 mass ratio) was placed at the closed end of a quartz tube. The substrates as prepared above are also placed inside the quartz tube at a distance from the precursor powders as seen in Fig. A1. A mixture of 99.5% argon and 0.5% oxygen gas is introduced into the chamber at 100 sccm and the chamber pressure allowed to stabilize at a pressure of 2.0 mbar. The tube furnace is then heated to 850 or 900 °C for 5 hours before allowing for cooling to room temperature. NWs are grown at the respective temperatures for 5 hours under a controlled gas pressure of 2 mbar.

To synthesize ZnO NWs with different optical properties, two parameters are varied. To grow ZnO NWs with green fluorescence, the tube furnace is heated to 900 °C and the substrate is positioned at 24 cm away from the source material. To grow ZnO NWs with orange-red fluorescence, the tube furnace is heated to 850 °C and the substrate is placed 21 cm from the source material. Details can be found in an earlier publication.

### Field emission measurements

The sample was mounted on an aluminium plate which served as the cathode. A glass microscope slide coated with indium tin oxide (ITO) served as the anode the ITO coating allowing the glass slide to be conductive yet transparent. The separation of the sample from the anode was controlled by the number of plastic spacers, in increments of 100 *µm*. The field emission experiment was carried out at <5 × 10^−6^ mbar. The anode and cathode of the sample stage were connected via electrical feedthroughs to a Keithley 237 High Voltage Source- Measurement Unit (SMU). It was used as both a voltage source as well as the measuring device.

### Scanning electron microscopy

A field emission Scanning Electron Microscope (JEOL JSM-6700F) was used to characterize the size and morphology of the ZnO:C NWs before and after field emission.

### Photoluminescence

A He-Cd laser centred at 325 nm was used as an excitation source for micro-photoluminescence (Renishaw inVia) measurements.

Kelvin probe microscopy was performed using a Bruker scanning atomic force microscope in frequency (FM) mode. Both the topography and surface potential were measured concurrently and the feedback loops were employed accordingly. We grounded the gold films with deposited ZnO-based NWs by attaching carbon tape onto the top surface of the gold film and connecting the other end of the circuit to the sample plate and grounding cable. An Al-coated silicon probe with a resonant frequency of approximately 330 kHz and spring constant of approximately 0.8 Nm^−1^ (band of cantilever) was used for the measurements. The gold substrate surface potential is used as the standard during measurement for comparison, similar to other works^21^.

X-ray photoelectron spectroscopy (Mg-Kα: 1253.6 eV) was also carried out on the samples. C1s peak from adventitious carbon at 285.0 eV is used as reference for shift correction due to charging. Photoelectrons were taken at a normal take-off angle relative to the surface plan.

### Fluorescence microscopy

A fluorescence microscope (based on Olympus-BX 51 coupled with both a halogen lamp and a mercury lamp) was used to capture images of the ZnO NWs under both bright field and ultraviolet (UV) illumination.

### High –resolution transmission electron microscopy

The samples were observed with high resolution transmission electron microscopy (HRTEM), JEOL JEM-3010 under both bright field (BF) imaging and selected area electron diffraction (SAED) modes.

### Post-processing: annealing

Annealing of ZnO NWs was carried out in ambient conditions in a Carbolite tube furnace at 850 °C.

The data generated during and/or analysed during the current study are available from the corresponding author on reasonable request.

## Supplementary information

Supporting Information
