# Supplementary material for: Fluorescence Invigoration in Carbon-Incorporated Zinc Oxide Nanowires from Passage of Field Emission Electrons
Source: Sci Rep. 2019 Jul 4;9:9671. doi: 10.1038/s41598-019-46177-w (PMC6609609; doi:10.1038/s41598-019-46177-w)
Supplement: Supplementary file 1 — Supporting Information [file 41598_2019_46177_MOESM1_ESM.docx]

**Supplementary Information**

**Fluorescence Invigoration in Carbon-Incorporated Zinc Oxide Nanowires from Passage of Field Emission Electrons**

*Andrew Bah,^1#^ Kim Yong Lim,^1#^ Fuhua Wei,^1^ Anjam Khursheed,^2^ Chorng Haur Sow^1,*^*

^1^Department of Physics, National University of Singapore, 2 Science Drive 3, Singapore 117542, Singapore

^2^Department of Electrical and Computer Engineering, National University of Singapore, 4 Engineering Drive 3, Singapore 117583, Singapore

# Equal contribution

* Correspondence and requests for materials should be addressed to C.H. Sow (physowch@nus.edu.sg)


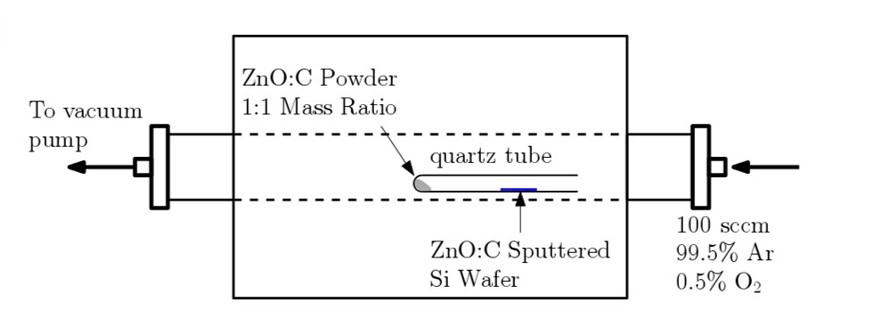


**Figure A1** Schematic diagram of tube furnace used for the CVD growth of the ZnO NWs, showing the placement of the ZnO:C powder mixture and ZnO-sputtered Si wafer within a quartz tube inside the tube furnace.

**
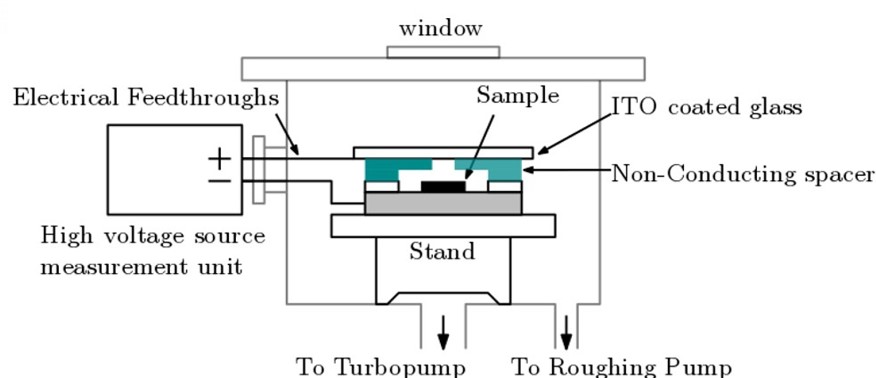
**

**Figure A2** Diagram showing the setup used for field emission testing of the ZnO nanowire sample.

The spacer acted to ensure that there was no electrical contact between the sample and the anode, as well as to define the area of the sample exposed to the anode. The sample, spacer and anode ITO glass were clamped to the cathode to achieve good electrical contact of the sample to the cathode, as well as to ensure minimum separation between the sample and the anode. The sample stage was placed in a chamber which was then vacuum pumped using a mechanical and turbomolecular pump to achieve a vacuum pressure of *<* 5 *×* 10*^−^*^6^ mbar after several hours of pumping.


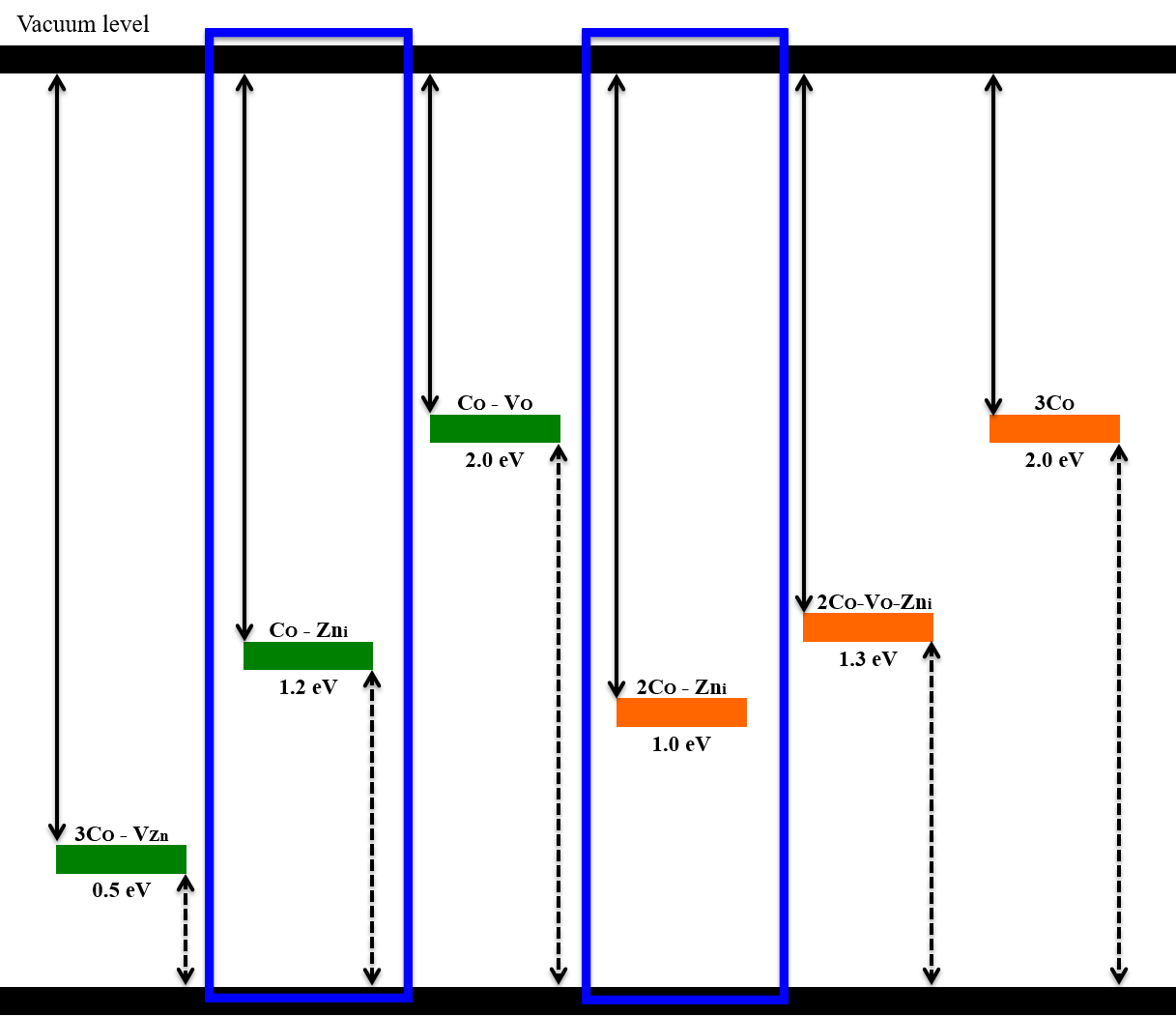


**Figure A3** Proposed pathways of work function of a variety of defects as computed and predicted from previous DFT calculations.^8,23^

Three possible defects contribute to green fluorescence, which are marked by horizontal green lines; the other three horizontal orange-red lines indicate three possible defects that contribute to orange-red fluorescence. The horizontal black line at the bottom presents the Fermi level of pure bulk ZnO is regarded as the zero-reference line. The other horizontal black line at the top presents the vacuum level. Vertical solid black arrowed lines indicate differences in energy between the respective highest occupied defect levels and the vacuum level. The defects highlighted in blue boxes exhibit work functions of 0.2eV difference between defect responsible for orange-red fluorescence and green fluorescence.


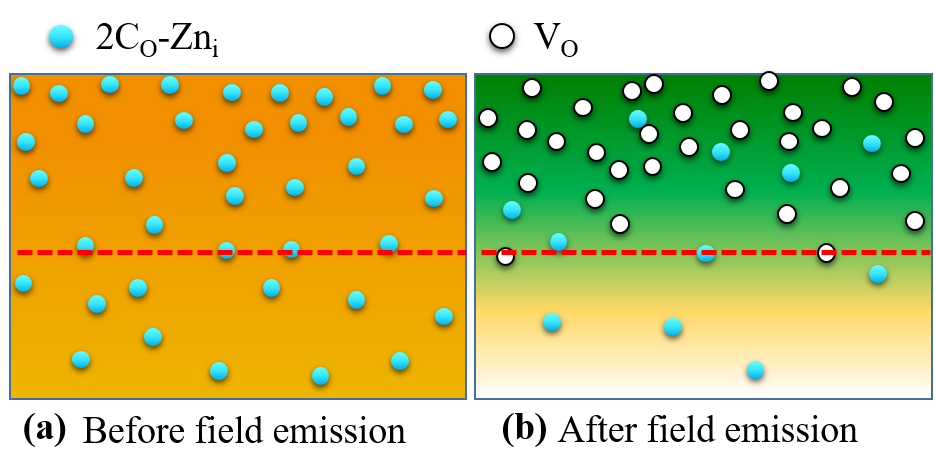


**Figure A4:** Schematics of proposed mechanism where (a) as-grown C-ZnO NWs with C-related defects and corresponding orange-red emissions. And (b) ZnO NWs after field emission with most of carbon-related defects removed and leaving behind more oxygen vacancies and the corresponding green fluorescence (~535nm) from regions closer to the NWs’ surface. The NWs’ cores are further made up of ordered ZnO upon diffusion of defects to the surface and this increases the excitonic photoluminescence (~380nm).

**(a)**





**(b)**





**Figure A5:** (a) PL spectra of as-grown NWs, NWs after field emission and NWs after field emission followed by plasma etching. The as-grown NWs show orange-red emission; the NWs after field emission show broadband spectral enhancement with green emission; the NWs after field emission followed by plasma etching show quenched visible wavelength emissions. (b) shows the respective normalised spectra.


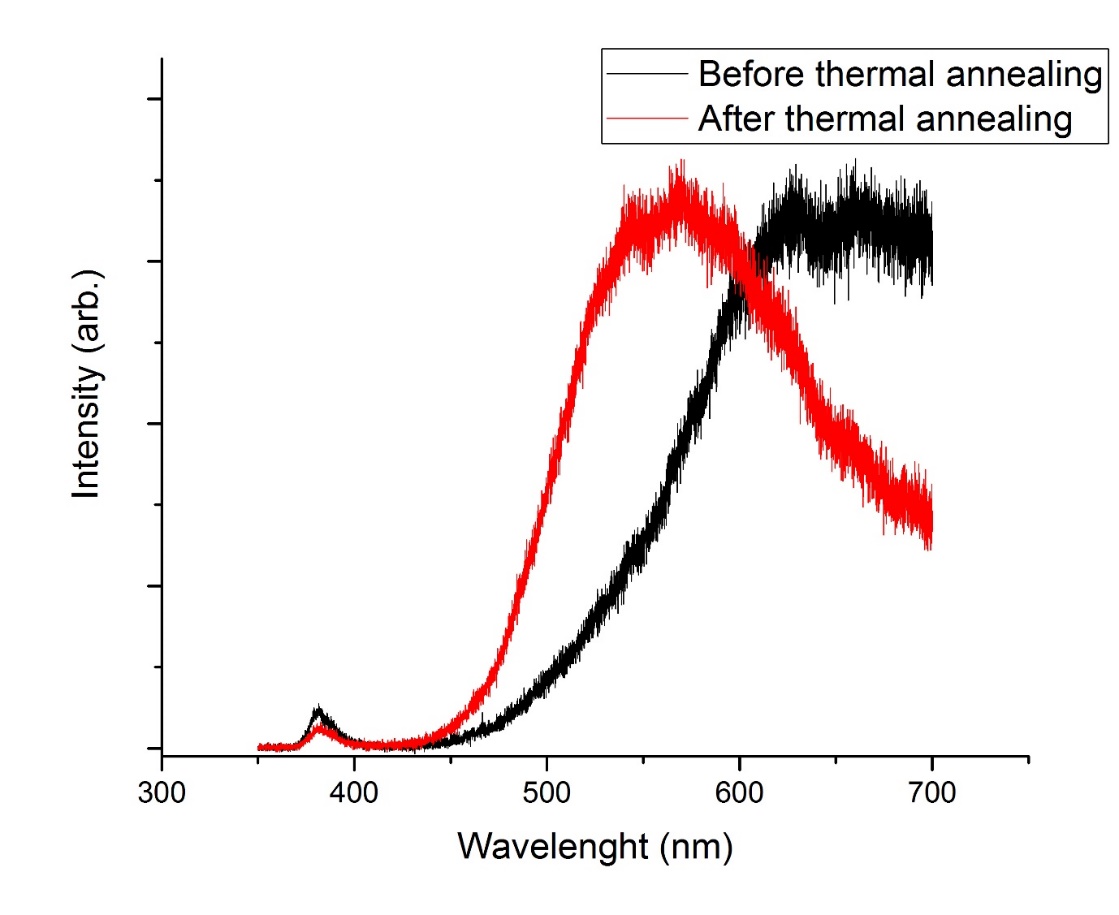


**Figure A6**: PL scans for Sample II (red-orange fluorescence) before and after thermal annealing in low vacuum, low oxygen conditions at 700*^◦^*C for 1 hour. After thermal annealing, defect peak has shifted from 600-700nm to around 550nm. Ratio of exciton to defect peak ratio is also reduced after field emission. Note that this data has not been normalized, and that the peak intensity before and after shows little variation, as compared to the increase in fluorescence intensity shown in Figure 6(e).
